# Interaction between *apolipoprotein E* genotype and hypertension on cognitive function in older women in the Nurses’ Health Study

**DOI:** 10.1371/journal.pone.0224975

**Published:** 2019-11-07

**Authors:** Iris Y. Kim, Francine Grodstein, Peter Kraft, Gary C. Curhan, Katherine C. Hughes, Hongyan Huang, Jae H. Kang, David J. Hunter

**Affiliations:** 1 Department of Epidemiology, Harvard T. H. Chan School of Public Health, Boston, Massachusetts, United States of America; 2 Nuffield Department of Population Health, University of Oxford, Oxford, United Kingdom; 3 Department of Medicine, Channing Division of Network Medicine, Brigham and Women’s Hospital and Harvard Medical School, Boston, Massachusetts, United States of America; 4 Department of Biostatistics, Harvard T. H. Chan School of Public Health, Boston, Massachusetts, United States of America; 5 Department of Nutrition, Harvard T. H. Chan School of Public Health, Boston, Massachusetts, United States of America; Nathan S Kline Institute, UNITED STATES

## Abstract

**Objective:**

To examine the interaction between APOE genotypes and both treated and untreated hypertension on cognitive function in an updated analysis of Nurses’ Health Study (NHS) data.

**Design:**

At baseline (1995–2001) and 3 biennial follow-up assessments over ~6 years, cognitive function was assessed.

**Setting and participants:**

8300 NHS participants aged 70+ years underwent a cognitive battery, which comprised 6 tests including the Telephone Interview for Cognitive Status (TICS) and tests of verbal memory, category fluency, and working memory.

**Measures:**

We estimated the mean differences in average cognitive scores across up to 4 assessments using multiple linear regression. We also tested for interaction between APOE e4 allele carrier status and hypertension overall, as well as for apparently untreated and treated hypertension.

**Results:**

We confirmed that, compared with those with APOE e3/3 genotype, APOE e4 allele carriers scored lower by 0.55 units on the average TICS score (95%CI:-0.67,-0.43). We also observed a significantly worse average TICS score among women with untreated hypertension compared with women without hypertension (difference = -0.23, 95%CI:-0.37,-0.09), while no significant difference was observed for women with treated hypertension. Significant interaction was detected between the APOE e4 allele and untreated hypertension (*p*-int = 0.02 for the TICS; *p*-int = 0.045 for global score), but not with treated hypertension. Specifically, compared with normotensive women with the APOE e3/3 genotype, APOE e4 allele carriers with treated hypertension scored lower by 0.50 units (95%CI:-0.69,-0.31); however, the APOE e4 allele carriers with untreated hypertension scored lower by 1.02 units on the TICS score (95%CI:-1.29, -0.76). This interaction of APOE e4 and untreated hypertension was also consistently observed for the global score.

**Conclusions:**

Women with hypertension and at least one APOE e4 allele had worse average cognitive function compared with women without hypertension with the e3/3 genotype; this difference was amplified among APOE e4 allele carriers with untreated hypertension.

## Introduction

The *apolipoprotein E (APOE) e4* allele is a well-established genetic risk factor for cognitive impairment and late-onset Alzheimer’s disease (AD) [1–5]. Genetic testing for *APOE e4* is currently not recommended, largely because of the perception that there are no interventions that reduce risk [6].

Vascular risk factors, such as hypertension, have also been associated with poor cognitive function [7] and an increased risk of dementia [8]. Hypertension is a modifiable risk factor. Studies of the interaction between genetic factors (*APOE e4*) and hypertension on cognitive function have been conducted [9–19]. However, results have been inconsistent, possibly due to insufficient power and the inability to distinguish controlled from uncontrolled hypertension. In addition, few studies have examined whether antihypertensive medication use influences the association between hypertension and *APOE e4* genotype on late-life cognitive function [9,12,14].

Results from the Nurses’ Health Study (NHS) have been published previously [11]; we present here updated results with almost twice the number of participants with *APOE e4* genotype and longer follow-up. With 8,300 participants who have completed up to four cognitive assessments, we evaluated the combined effects of the *APOE e4* genotype and history of physician-diagnosed hypertension on long-term cognitive function in a large longitudinal cohort of female nurses.

## Methods

### Study population

In 1976, the Nurses’ Health Study began when 121,700 female registered nurses of ages 30–55 returned a mailed, self-administered questionnaire about their lifestyle and medical history. Similar questionnaires were collected biennially thereafter with >90% follow-up rate [20]. The nurses were predominantly of European descent (97% White), which reflects the racial composition of nurses at the time of recruitment.

From 1995–2001, a subcohort of women aged 70 years and older without diagnosed stroke was selected to participate in a telephone-based study of cognitive function. Among the 22,213 eligible women, 93% participated in the first interview (n = 19,415). Three additional follow-up assessments were performed every two years until June 2008.

To obtain genetic data, blood samples were collected from participants in 1989–1990. Among those who had not provided blood samples, participants were invited to provide buccal cell samples from 2002–2004. The baseline cohort for this study is comprised of the 9,172 women who provided a sample and were genotyped. We excluded participants with the e4/e2 genotype since we examined the associations for the *APOE e4* and *e2* allele separately (n = 165). In addition, we further excluded women without information on history of hypertension (n = 33) and without at least one complete battery of cognitive assessments (n = 674). Therefore, our analytical sample included 8,300 women.

### Laboratory analyses

Genomic DNA was extracted using the ReturPureGene DNA Isolation Kit (Gentra Systems Minneapolis, MN), which was then genotyped using a Taqman assay (Applied Biosystems, Foster City, CA) [11, 16], or *APOE* allele status was imputed from a GWAS chip [21]. Laboratory personnel were blinded to participants’ medical history and cognitive function assessments.

### Hypertension and other covariate assessment

Information on physician-diagnosed hypertension was collected every two years by questionnaires. The validity of participants’ self-reported diseases and health outcomes compared with their medical records has been previously reported with high accuracy [22–24]. In our analyses, we considered history of hypertension as any self-report of physician-diagnosed hypertension from 1976 to the biennial questionnaire immediately before each participant’s first cognitive assessment. Information on medications used for high blood pressure treatment (e.g., beta blockers, calcium channel blockers, ACE inhibitors, thiazide diuretics) was collected every two years, as well as information on lifestyle characteristics, such as smoking status and physical activity.

### Cognitive function assessment

Assessment of cognitive function via validated telephone interviews has been previously described in detail [25]. Briefly, trained interviewers administered the initial cognitive interview using only the Telephone Interview of Cognitive Status (TICS) [26], an adaptation of the Mini-Mental State Exam (MMSE). We gradually added five other cognitive tests: 2) immediate and 3) delayed recalls of the East Boston Memory test (EBMT), 4) delayed recall of the TICS 10-word list, 5) category fluency; and 6) digit span-backward. Because the range of scores varied across tests and points are not equivalent for each test, we standardized the scores by calculating the z-scores of each test and then taking average of these z-scores to calculate the global score. A composite verbal memory score was also derived by averaging test-specific z-scores of the immediate and delayed recalls of the EBMT and TICS 10-word list. The primary outcomes for our analyses were the TICS and the composite global score.

The validity and reliability of the telephone interviews have been previously established [25]. In the NHS, the correlation comparing the TICS score from the telephone interview with an in-person interview was 0.81. In addition, there were high correlations for scoring of each test in a study of inter-rater reliability (r = 0.95).

### Statistical analyses

#### Analyses of average cognitive function by APOE genotypes

For our primary analyses, we used multiple linear regression to estimate multivariable-adjusted mean differences and corresponding 95% CI of average scores of up to four cognitive assessments by *APOE* genotypes. Using the average of repeated assessments minimizes variability and measurement error in determining cognitive performance. Because it is more difficult to detect stable measurement of cognitive decline over a relatively short follow-up period, we estimated the rate of annual change in sensitivity analyses. For all analyses, participants with the *e3/e3* genotype served as the reference group. We performed tests of linear trend across increasing number of *APOE e4* alleles (*e3/3*, *e3/4*, and *e4/4*) and *APOE e2* alleles (*e3/3*, *e3/2*, and *e2/2*). To assist in understanding the clinical significance of the results, we also estimated the mean differences in scores of *e4* carriers compared with *e3/3* carriers associated with a one-year increase in age.

The multivariable-adjusted models adjusted for the following potential confounders, which were time-updated until the study baseline: age at interview (years), highest attained education (Registered Nurse diploma, Bachelor’s degree, or Master’s or Doctoral degree), history of high cholesterol (yes/no), cigarette smoking (current, past, never), alcohol intake (0, 1–4, 5–14, ≥15 g/day), body mass index (<22, 22–24.9, 25–29.9, ≥30 kg/m^2^), physical activity (quintiles of metabolic equivalents-hour/week), current aspirin use (0, 1–2 days/week, ≥3 days/week), other NSAID use (yes/no), current vitamin E supplement use (yes/no), postmenopausal hormone use (never, current, past), antidepressant use (yes/no), mental health index (0–51 [depressed], 52–100 [not depressed]), and energy-fatigue index (0–49 [low vitality], 50–100 [high vitality]) from the Medical Outcomes Short Form-36 (SF-36).

#### Analysis of interaction between APOE e4 genotype and physician-diagnosed hypertension

To evaluate the joint effects of *APOE e4* genotypes and hypertension on average cognitive function, we used four exposure categories: having *e3/3* genotype and hypertension, having at least one e4 allele but no hypertension, having at least one *e4* allele and hypertension (category with lowest expected cognitive performance), and the reference group of having *e3/3* genotype and no hypertension (category with highest expected cognitive performance). We performed tests of multiplicative interaction between *APOE e4* genotype and history of hypertension by testing the significance of the product term between the two variables. In addition, we further stratified the group of women who had at least one *e4* allele and history of hypertension by anti-hypertensive medication use to determine whether there was an interaction between the *APOE e4* genotype and treated/untreated hypertension on average cognitive performance.

#### Sensitivity analyses

We examined the change in scores between participants with at least one *e4* allele (i.e., *e3/e4* or *e4/e4*) compared with those with *e3/e3* genotype over four interviews (i.e., 6 years). Multivariable-adjusted linear mixed models were used to account for the correlations in participant’s observations across the assessments with a “robust baseline,” in which we averaged the scores of the first two cognitive assessments to adjust for possible learning effects at the second cognitive assessment. We adjusted for the same covariates as in the analyses for average cognitive function. Similar analyses were performed to assess the mean rate of cognitive decline comparing women with a history of physician diagnosed hypertension compared with women with no history of hypertension.

In other secondary analyses, we considered different definitions of hypertension used in previous studies [27,28], including dichotomizing hypertension based on self-reports of systolic blood pressure (SBP) at ≥145+ mmHg compared with <145 mmHg and considering SBP in three categories: <105–124 mmHg (reference), 125–154 mmHg, ≥155 mmHg. In addition, we also classified hypertension as having a SBP≥145 mmHg or diastolic blood pressure of ≥90 mmHg. Finally, to determine the robustness of our results, we also compared average cognitive function at baseline and used multivariable logistic regression to calculate odds ratio of a “low” cognitive function score (i.e., scoring at the bottom 10^th^ percentile).

## Results

Baseline characteristics of the NHS cohort according to categories of physician-diagnosed hypertension and *APOE e4* genotype are presented in Table 1. As expected, nurses with *APOE e4* genotype reported having history of high cholesterol more frequently compared to *e3/e3* carriers. Women with a history of hypertension were more likely to be obese compared to women with no history of hypertension. The distribution of other predictors of poor cognitive function was similar across the exposure categories. We report participants’ characteristics across exposure categories further stratified by treated and untreated hypertension in S1 Table and the distribution of cognitive function scores in S2 Table.

**Table 1:** Age-adjusted characteristics of women at baseline^a^ according to physician-diagnosed hypertension and APOE e4 genotype status (n = 8300).

	Physician-diagnosed hypertension and APOE e4 status			
	HTN^b^-, e4- (n = 2931)	HTN+, e4- (n = 3525)	HTN-, e4+ (n = 833)	HTN+, e4+ (n = 1011)
Age, years*	74.1(2.2)	74.4(2.3)	74.0(2.2)	74.3(2.2)
Masters/doctorate degree (%)	7.0	6.0	5.0	5.9
History of high serum cholesterol (%)	56.4	70.5	66.0	79.0
Obesity (body mass index > 30 kg/m^2^) (%)	12.1	24.4	10.2	21.3
Self-perceived low energy (<50 in SF-36 energy/fatigue index) (%)	12.9	19.8	12.1	15.2
Self-perceived depression (<52 in SF-36 mental health index) (%)	3.3	4.3	4.0	4.1
Current regular aspirin use (%)	52.4	58.0	56.7	56.9
Current regular other NSAIDs use (%)	16.9	20.1	16.6	18.1
Current Vitamin E use (%)	50.6	51.4	51.5	51.8
Current postmenopausal hormone use (%)	36.4	34.7	36.0	37.8
Current smoker (%)	8.9	5.5	8.3	8.3
Past smoker (%)	43.7	47.2	44.5	47.2
Mean physical activity, MET-h/week	18.3(20.3)	14.8(17.6)	18.6(22.1)	15.0(17.9)
Mean alcohol intake, g/day	4.9(9.2)	4.5(8.8)	5.4(9.0)	5.2(9.9)

Values are means(SD) or percentages and are standardized to the age distribution of the study population

^a^ All characteristics represent those reported from the biennial questionnaire immediately prior to participants’ baseline telephone interviews (1995–2001)

^b^ Abbreviations: HTN = physician diagnosed hypertension; APOE e4 = apolipoprotein E e4 allele; e4+ = APOE e4 allele carrier; e4- = non-APOE e4 allele carrier; NSAIDs = non-steroidal anti-inflammatory drugs; SF-36 = Short Form 36; MET-h/week = metabolic equivalents of task-hours per week

* Value is not age-adjusted

**Fig 1:**
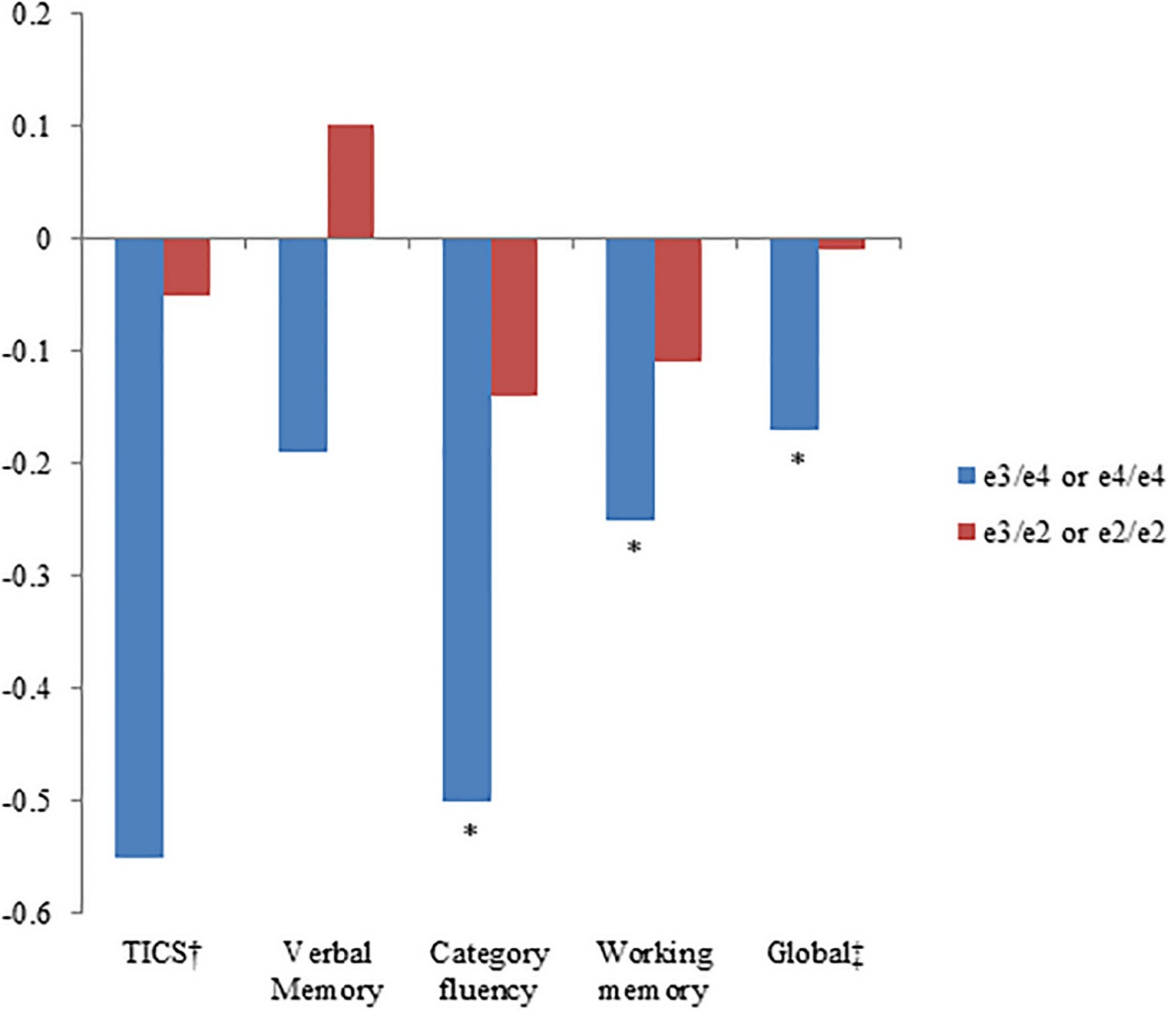
Multivariable-adjusted mean differences in average cognitive performance in up to 4 assessments according to apolipoprotein E genotypes compared to the e3/e3 genotype. Multivariable models adjusted for age, education, high blood pressure, high cholesterol, cigarette smoking, alcohol intake, body mass index, physical activity, aspirin use, other NSAIDs use, Vitamin E supplement use, postmenopausal hormone use, antidepressant use, mental health index and energy-fatigue index from SF-36. *P<0.0001. ^†^Telephone Interview of Cognitive Status. ‡Global score is the average of the z-scores of TICS, immediate and delayed recalls of East Boston Memory Test, delayed recall of TICS 10-word list, test of category fluency, digit backwards test.

Approximately 64.8% of the participants carried the *e3/3* genotype (reference) while 22.2% had at least one *e4* allele and 13.0% had at least one *e2* allele. The coefficient of variation was greater than 10% of for some of the cognitive test scores that assess specific cognitive domains (i.e., category fluency and working memory) compared to the tests that assess overall cognitive performance (i.e., TICS), which was expected. Fig 1 shows the main effect of *APOE e4* and *e2* genotypes on average cognitive performance over up to 4 assessments.

As expected, across all cognitive tests, *e3/e4* genotype carriers had lower average cognitive scores compared to homozygous *e3* carriers, and homozygous *e4* carriers had even lower average cognitive scores compared to homozygous *e3* carriers. Models adjusting for additional covariates produced similar results compared to models only adjusting for age and education. On the TICS score, nurses with a *e3/4* or *e4/e4* genotype scored lower by 0.55 units on average compared to those with *e3/3*. On the same score, women 1 year older scored lower on average by 0.20 points; therefore, we considered the effect of having an *e4/e4* genotype equivalent to being older by almost 3 years on average. There was a strong dose response relationship per increasing e4 allele among those with *e3/3*, *e3/4*, and *e4/4* (*p*_trend_<0.0001 for all cognitive assessments). We did not find significant mean differences in any of the cognitive scores by *APOE e2* genotypes. Women with treated hypertension did not perform significantly worse on cognitive assessments compared with women with no history of physician-diagnosed hypertension (Table 2). However, untreated hypertension was associated with lower average cognitive performance across several cognitive domains. Women with untreated hypertension scored 0.23 units lower on the TICS compared to women with no hypertension (95% CI: -0.37, -0.09; *p* = 0.002); this is cognitively equivalent to the mean difference in cognitive performance associated with 1.21 years of aging (0.19 decrease in unit per 1 year of aging, *p*<0.0001). When examining the effect of antihypertensive medication use among women with hypertension, untreated participants had an average score of 0.17 units lower on the TICS compared to treated participants (95%: -0.31, -0.03; p = 0.02). There was no significant difference on the global score between treated and untreated hypertensive participants.

**Table 2:** Multivariable-adjusted mean differences (95% CI) in average cognitive performance in up to 4 assessments (1995–2008), by history of physician-diagnosed hypertension (n = 8300; 1995–2008), with and without treatment.

	**TICS**^a^	**Verbal memory**	**Category fluency**	**Working memory**	**Global**^b^
No hypertension (n = 3764)	REF	REF	REF	REF	REF
Treated hypertension (n = 3194)					
Age, education-adjusted difference in score (95% CI)	-0.09 (-0.19, 0.02)	-0.01 (-0.03, 0.02)	-0.30 (-0.48, -0.11)	-0.09 (-0.18, -0.001)	-0.02 (-0.04, 0.01)
Multivariable-adjusted mean difference (95% CI)^c^	-0.05 (-0.16, 0.06)	-0.00 (-0.03, 0.03)	-0.27 (-0.46, -0.09)	-0.06 (-0.15, 0.03)	-0.01 (-0.04, 0.02)
Untreated hypertension (n = 1342)					
Age, education-adjusted difference in score (95% CI)	-0.31 (-0.45, -0.17)	-0.05 (-0.09, -0.01)	-0.32 (-0.56, -0.08)	-0.15 (-0.26, -0.03)	-0.06 (-0.09, -0.02)
Multivariable-adjusted mean difference (95% CI)^c^	-0.23 (-0.37, -0.09)	-0.04 (-0.08, 0.00)	-0.24 (-0.48, 0.00)	-0.10 (-0.22, 0.02)	-0.04 (-0.08, -0.004)
Mean difference in score associated with 1 year of aging	-0.19 (-0.22, -0.17)	-0.05 (-0.05, -0.04)	-0.24 (-0.28, -0.20)	-0.07 (-0.09, -0.06)	-0.05 (-0.05, -0.04)
Clinical significance in years of aging^d^	1.21	0.80	1.00	1.43	0.80
	**TICS**^a^	**Verbal memory**	**Category fluency**	**Working memory**	**Global**^b^
Untreated hypertension vs. treated hypertension (REF)(n = 4536)	-0.17 (-0.31, -0.03)	-0.03 (-0.07, 0.01)	0.06 (-0.19, 0.31)	-0.05 (-0.17, 0.07)	-0.03 (-0.06, 0.01)

^a^ Telephone Interview of Cognitive Status.

^b^ Global score is the average of the z-scores of TICS, immediate and delayed recalls of East Boston Memory Test, delayed recall of TICS 10-word list, test of category fluency, digit backwards test.

^c^ Multivariable models adjusted for age, education, high blood pressure, high cholesterol, cigarette smoking, alcohol intake, body mass index, physical activity, aspirin use, other NSAIDs use, Vitamin E supplement use, postmenopausal hormone use, antidepressant use, mental health index and energy-fatigue index from SF-36.

^d^ To help interpret the mean differences in scores, we provide the mean difference in performance associated with 1 year of aging. For example, women 1 year older scored lower by 0.05 points for the mean global performance across 4 assessments, adjusting for the aforementioned covariates; thus the -0.04 points difference for self-reported untreated hypertension compared to having no hypertension can be considered equivalent to being older by approximately 0.80 years.

**Fig 2:**
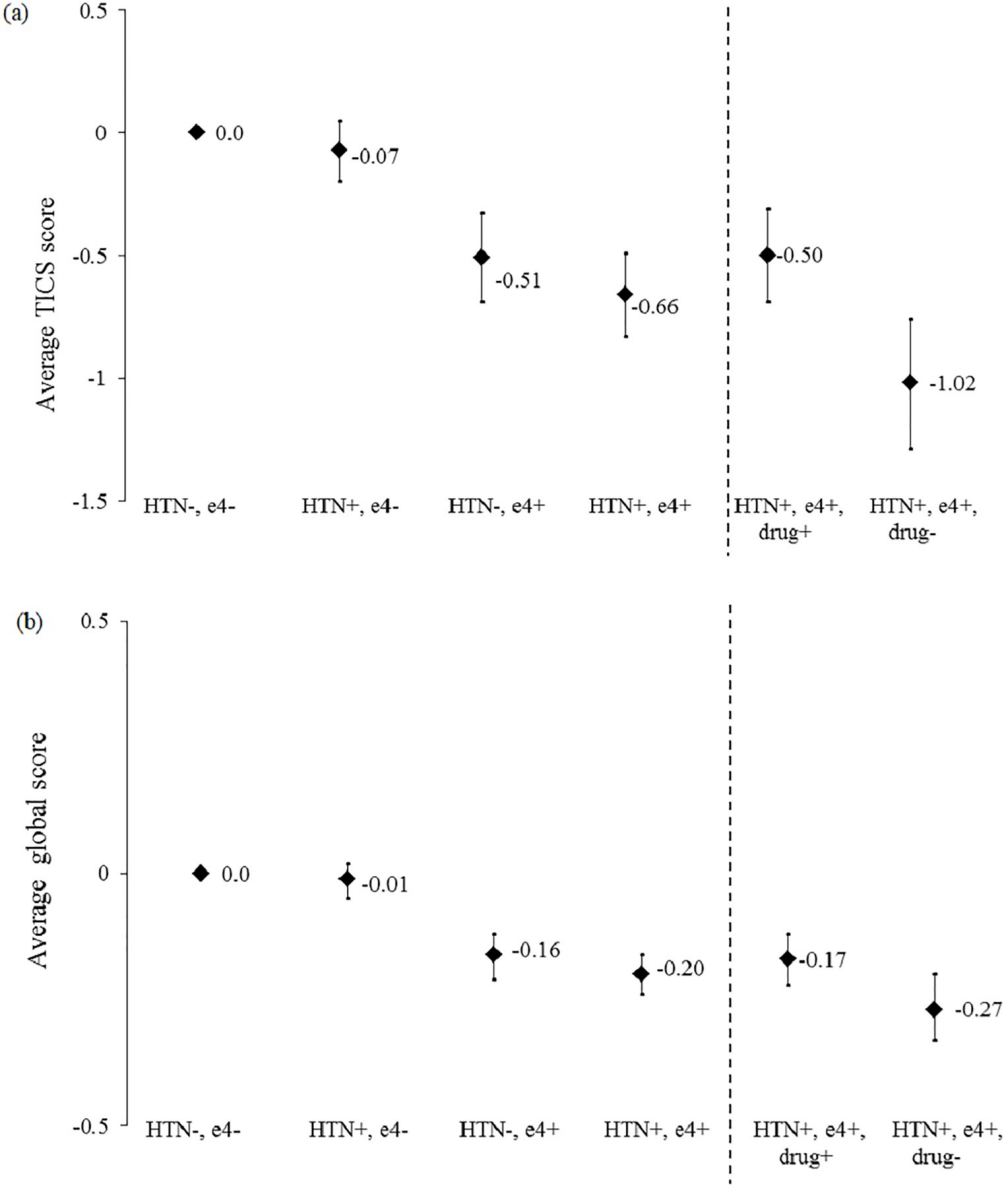
Adjusted mean differences in (a) average TICS score and (b) average global score according to categories of physician-diagnosed hypertension (“HTN”) and apolipoprotein e4 genotypes. *P*-for interactions between APOE e4 and treated HTN were 0.61 and 0.71, respectively; *P*-for interactions between APOE e4 and untreated HTN were 0.02 and 0.045, respectively.

Results of the joint effects of *APOE e4* genotype and history of physician-diagnosed hypertension, with and without treatment, on average cognitive scores are presented in Table 3. Overall, the subgroup of *e4* carriers with untreated hypertension performed the worst on the cognitive assessments. Compared with the referent group without hypertension and *e3/e3* carriers, women with untreated hypertension who had at least one *APOE e4* allele scored 1.02 units lower on the TICS (95% CI: -1.29, -0.76; *p*<0.0001), which is cognitively equivalent to the effect of aging by 5.1 years (-0.20 units per 1 year of aging, 95% CI: -0.22, -0.18; *p*<0.0001) (Fig 2a). In general, among participants who were both hypertensive and carriers of the *APOE e4* allele, those who did not use antihypertensive drugs performed substantially worse than those who used antihypertensive drugs. However, women with treated hypertension had average cognitive scores that were comparable to women without a history of hypertension among carriers of the *APOE e4* genotype.

**Table 3:** Multivariable-adjusted* mean differences (95% CI) in average cognitive performance in up to 4 assessments (1995–2008), by APOE e4 status an history of physician-diagnosed hypertension (n = 8300; 1995–2008), with and without treatment.

	N	TICS^a^	Verbal memory	Category fluency	Working memory	Global^b^
Physician-diagnosed hypertension						
HTN-, e4-^¶^ (REF)	2931	REF	REF	REF	REF	REF
HTN+, e4-^¶^	3525	-0.07 (-0.20, 0.05) P = 0.25	-0.01 (-0.05, 0.02) P = 0.50	-0.21 (-0.42, 0.01) P = 0.06	-0.04 (-0.14, 0.07) P = 0.50	-0.01 (-0.05, 0.02) P = 0.35
HTN-, e4+^¶^	833	-0.51 (-0.69, -0.33) P<0.0001	-0.19 (-0.24, -0.14) P<0.0001	-0.44 (-0.74, -0.13) P<0.01	-0.17 (-0.32, -0.02) P = 0.03	-0.16 (-0.21, -0.12) P<0.0001
HTN+, e4+^¶^	1011	-0.66 (-0.83, -0.49) P<0.0001	-0.21 (-0.25, -0.16) P<0.0001	-0.77 (-1.06, -0.48) P<0.0001	-0.36 (-0.50, -0.22) P<0.0001	-0.20 (-0.24, -0.16) P<0.0001
Treatment+^¶^	701	-0.50 (-0.69, -0.31) P<0.0001	-0.17 (-0.22, -0.12) P<0.0001	-0.80 (-1.13, -0.47) P<0.0001	-0.27 (-0.43, -0.10) P<0.01	-0.17 (-0.22, -0.12) P<0.0001
Treatment-^¶^	310	-1.02 (-1.29, -0.76) P<0.0001	-0.29 (-0.36, -0.21) P<0.0001	-0.70 (-1.16, -0.23) P<0.01	-0.57 (-0.79, -0.34) P<0.0001	-0.27 (-0.33, -0.20) P<0.0001
p-interactions^c^		P = 0.61 for treatment + P = 0.02 for treatment -	P = 0.44 for treatment + P = 0.08 for treatment -	P = 0.53 for treatment + P = 0.85 for treatment -	P = 0.71 for treatment + P <0.01 for treatment -	P = 0.71 for treatment + P = 0.045 for treatment -

*Adjusted for age, education, high cholesterol, cigarette smoking, alcohol intake, body mass index, physical activity, aspirin use, other NSAIDs use, Vitamin E supplement use, postmenopausal hormone use, antidepressant use, mental health index and energy-fatigue index from SF-36.

^¶^HTN = physician diagnosed hypertension, Treatment+ = treated with anti-hypertensives, Treatment- = never reported treatment with anti-hypertensives, e4+ = APOE e4 allele carrier; e4- = non-APOE e4 allele carrier

^a^ Telephone Interview of Cognitive Status.

^b^ Global score is the average of the z-scores of TICS, immediate and delayed recalls of East Boston Memory Test, delayed recall of TICS 10-word list, test of category fluency, digit backwards test.

^c^ Interaction between APOE e4 and treated/untreated HTN

There was evidence of supra-multiplicative interaction between the presence of an *APOE e4* allele and untreated hypertension on several cognitive assessments (*p* = 0.02 for the TICS; *p* = 0.045 for global composite score; *p*<0.01 for working memory).

Results were similar in additional analyses examining the annual rate of cognitive decline, defining hypertension as having a SBP of at least 145 mmHg and in three categories, assessing cognitive scores at baseline, and conducting logistic regression to estimate scoring at the bottom 10^th^ percentile in cognitive performance.

## Discussion

### Statement of principal findings

In this longitudinal study of 8,300 women, as expected, the *APOE e4* allele was highly associated with decreased cognitive function compared with *e3/e3* genotype across all domains (*p* = 0.001). In addition, we found a strong dose-response with increasing number of e4 alleles (*p*_trend_ <0.0001).

We observed that untreated hypertension was associated with lower TICS score (*p* = 0.001) and lower global composite score (*p* = 0.03) compared with having no history of hypertension. These results are consistent with the suggested association between hypertension and increased risk of cognitive impairment and Alzheimer’s disease [19,29–36], particularly with the studies showing higher risks with untreated hypertension and lower risks with anti-hypertensive treatment. These studies suggest since high blood pressure increases risk of cognitive decline and dementia, possibly through development of ischemic white matter lesions in the brain [19,37], controlling hypertension may reduce risk.

### Results in relation to other studies

Although numerous studies suggest potential interactions between various vascular conditions and *APOE e4* on cognitive decline [13,38–40], few have examined interactions with high blood pressure, particularly with consideration to antihypertensive medication use [12,14]. Peila et al. (2001) reported that among participants with untreated high systolic blood pressure, *e4* carriers had the highest risk of poor cognitive function compared with those with normal SBP and *e3/3* carriers [14]. This association was not found among treated participants and three-way interaction with *e4* allele, high SBP, and antihypertensive treatment was borderline significant (p = 0.09). In our previous study in the NHS, we detected a significant interaction between untreated hypertension and *e4* genotype in tests of working memory (p<0.05); with longer follow up and increased power, we detected the same interactions in tests of working memory, as well as on the TICS and global composite score. However, the combined detrimental effects with *APOE e4* genotype and high blood pressure was attenuated among *e4* carriers who had their hypertension treated, consistent with results from Qui et al. (2003), who reported that the joint effect of *APOE e4* genotype and high systolic pressure was diminished among participants who used antihypertensive drugs [12]. Interestingly, chronic hypertension has been reported to be associated with tau pathology among *APOE e4* carriers [41,42], suggesting a plausibility in mechanism through which hypertension may interact with *APOE e4* to influence cognitive function.

### Strengths and limitations of study

Our present study extends on our previous research by incorporating a longer follow up time with a high active follow-up rate, greater power, detailed information on antihypertensive medication use, and repeated administration of comprehensive assessments across multiple cognitive domains. Our study has several limitations. First, there may have been measurement error in cognitive function assessments, particularly among participants who may have decided to complete certain tests while omitting others. However, in sensitivity analyses, results remained unchanged when we restricted our analyses to participants who had at least one missing cognitive assessment. In addition, we used the averages of up to four cognitive scores, providing a more robust measure of cognitive function. Our measures of cognitive function do not distinguish between age-related decline, vascular dementia, and the early onset of AD. However, the end point of cognitive function summarizes the manifestation of all three potential pathologies. Measurement error of physician-diagnosed hypertension is expected to be non-differential with respect to cognitive assessment, thereby biasing our results towards the null. We did not measure blood pressure in person, and relied on history of hypertension medications to distinguish treated from untreated hypertension. As expected, hypertensive women who were treated had lower SBP compared to hypertensive women who were not treated. Finally, although we adjusted for many potential covariates, the presence of unmeasured confounding is possible in observational studies and cannot be disregarded. Because our study was restricted to female nurses, our results may have limited generalizability; future studies in other populations are warranted.

### Conclusion

In summary, these results suggest that treating hypertension may be protective against poor cognitive function in late-life, particularly among *e4* carriers. While treatment of hypertension is a universal recommendation, treatment of hypertension may be particularly important among carriers of the *APOE e4* allele, and can provide a rationale for more widespread testing for the *APOE e4* allele.

## Supporting information

S1 Table Age-adjusted characteristics of women at baseline^a^ according to physician-diagnosed hypertension, treatment status, and APOE e4 genotype (n = 8300). (DOCX)

S2 Table Distribution of cognitive function scores. (DOCX)
