# Supplementary material for: Interaction between apolipoprotein E genotype and hypertension on cognitive function in older women in the Nurses’ Health Study
Source: PLoS One. 2019 Nov 7;14(11):e0224975. doi: 10.1371/journal.pone.0224975 (PMC6837309; doi:10.1371/journal.pone.0224975)
Supplement: S1 Table — (DOCX) [file pone.0224975.s001.docx]

|  | Physician-diagnosed hypertension, treatment status, and APOE e4 genotype | | | | | |  |
| --- | --- | --- | --- | --- | --- | --- | --- |
|  | HTN^b^-,  e4- (n=2931) | HTN+, treatment+ e4- (n=2493) | HTN+, treatment-e4- (n=1032) | HTN-,  e4+ (n=833) | HTN+, treatment+ e4+ (n=701) | HTN+, treatment-, e4+ (n=310) |  |
| Age, years^*^ | 74.1(2.2) | 74.4(2.3) | 74.3(2.3) | 74.0 (2.2) | 74.3(2.2) | 74.2(2.2) |  |
| Masters/doctorate degree (%) | 7.0 | 6.2 | 5.6 | 5.0 | 5.9 | 5.8 |  |
| History of high serum cholesterol (%) | 56.4 | 72.6 | 65.3 | 66.0 | 81.3 | 74.3 |  |
| Obesity (body mass index > 30 kg/m²) (%) | 12.1 | 26.0 | 20.6 | 10.2 | 21.5 | 21.4 |  |
| Self-perceived low energy (<50 in SF-36 energy/fatigue index) (%) | 12.9 | 20.1 | 19.3 | 12.1 | 15.0 | 16.2 |  |
| Self-perceived depression (<52 in SF-36 mental health index) (%) | 3.3 | 4.2 | 4.4 | 4.0 | 3.6 | 5.4 |  |
| Current regular aspirin use (%) | 52.4 | 59.4 | 54.6 | 56.7 | 58.4 | 53.4 |  |
| Current regular other NSAIDs use (%) | 16.9 | 21.1 | 17.6 | 16.6 | 18.6 | 17.3 |  |
| Current Vitamin E use (%) | 50.6 | 51.9 | 50.3 | 51.5 | 52.1 | 51.4 |  |
| Current postmenopausal hormone use (%) | 36.4 | 36.1 | 31.3 | 36.0 | 39.3 | 33.9 |  |
| Current smoker (%) | 8.9 | 5.3 | 6.3 | 8.3 | 7.7 | 9.7 |  |
| Past smoker (%) | 43.7 | 48.0 | 45.3 | 44.5 | 50.4 | 40.2 |  |
| Mean physical activity, MET-h/week | 18.3 (20.3) | 14.7 (17.7) | 14.9 (17.2) | 18.6 (22.1) | 14.5 (17.7) | 16.2 (18.4) |  |
| Mean alcohol intake, g/day | 4.9(9.2) | 4.5(8.7) | 4.5(9.0) | 5.4(9.0) | 5.5(10.0) | 4.7(10.0) |  |
| Values are means(SD) or percentages and are standardized to the age distribution of the study population  ^*^ Value is not age adjusted  ^a^ All characteristics represent those reported from the questionnaire immediately prior to participants’ baseline telephone interviews (1995-2001).  ^b^ HTN=physician diagnosed hypertension; APOE e4=apolipoprotein E e4 allele; e4+= APE e4 allele carrier; e4-= non-APOE e4 allele carrier | | | | | | | |

**S1 Table.** Age-adjusted characteristics of women at baseline^a^ according to physician-diagnosed hypertension, treatment status, and APOE e4 genotype (n=8300)
