# Supplementary material for: Interaction between apolipoprotein E genotype and hypertension on cognitive function in older women in the Nurses’ Health Study
Source: PLoS One. 2019 Nov 7;14(11):e0224975. doi: 10.1371/journal.pone.0224975 (PMC6837309; doi:10.1371/journal.pone.0224975)
Supplement: S2 Table — (DOCX) [file pone.0224975.s002.docx]

**S2 Table.** Distribution of cognitive function scores

|  | N | Mean | Std Dev | Coefficient of Variation (%) |
| --- | --- | --- | --- | --- |
| TICS | 8300 | 33.82 | 2.33 | 6.88 |
| Verbal memory^a^ | 8300 | 0.01 | 0.63 | N/A |
| Category fluency | 8300 | 17.03 | 3.94 | 23.12 |
| Working memory | 8300 | 6.62 | 1.90 | 28.78 |
| Global^a^ | 8300 | -0.04 | 0.58 | N/A |

^a^ The coefficient of variation for the verbal memory score and global score are undefined as the mean for these scores is 0.
